# Posterior reversible encephalopathy syndrome secondary to acute post-streptococcal glomerulonephritis

**DOI:** 10.1590/0037-8682-0267-2023

**Published:** 2023-07-24

**Authors:** Nurhayat Yakut, Hamdi Murat Yildirim, Cihangir Akgun

**Affiliations:** 1 Istanbul Medipol University, Medipol Bahcelievler Hospital, Department of Pediatrics, Division of Pediatric Infectious Diseases, Istanbul, Turkey. Istanbul Medipol University Medipol Bahcelievler Hospital Department of Pediatrics Istanbul Turkey; 2 Medipol Bahcelievler Hospital, Department of Pediatrics, Division of Pediatric Intensive Care, Istanbul, Turkey. Medipol Bahcelievler Hospital Department of Pediatrics Division of Pediatric Intensive Care Istanbul Turkey; 3 Istanbul Medipol University, Medipol Bahcelievler Hospital, Department of Pediatrics, Division of Nephrology, Istanbul, Turkey. Istanbul Medipol University Medipol Bahcelievler Hospital Department of Pediatrics Istanbul Turkey

A previously healthy 11-year-old boy presented to the emergency department with generalized tonic-clonic seizures. One week prior, he had complained of headaches, blurry vision, nausea, and vomiting. Once the seizures were controlled with midazolam and levetiracetam, the patient was admitted to the pediatric intensive care unit. The patient had an altered mental status and macroscopic hematuria with a blood pressure of 190/120 mmHg. Initial laboratory testing revealed microscopic hematuria (161 red blood cell/µL) and proteinuria (0,6 gr/24 h), a low level of complement factor 3 (0.34 g/L), and an elevated anti-streptolysin O antibody titer (459 IU/mL). Cranial magnetic resonance imaging showed an abnormal signal and contrast enhancement in the right cerebellar hemisphere, consistent with the radiological diagnosis of posterior reversible encephalopathy syndrome (PRES) (Figures A, B, C). The patient was diagnosed with PRES following hypertensive encephalopathy related to acute post-streptococcal glomerulonephritis based on clinical, laboratory, and radiological findings and so he was started on intravenous esmolol, furosemide, and oral amlodipine. The patient’s encephalopathy had improved. His blood pressure was controlled with oral amlodipine, and the macroscopic hematuria resolved. The patient was discharged upon normalization of his neurological examination results, with the exception of occasional blurry vision. PRES is a clinical and radiological disorder of varying etiologies and its treatment is based on identifying and correcting the underlying condition^1^^,^^2^. This case highlights the rare association between PRES and acute post-streptococcal glomerulonephritis in a child. Owing to the irreversible negative consequences of PRES, early diagnosis and appropriate treatment are vital to provide good outcomes^3^. 

**FIGURE A:**
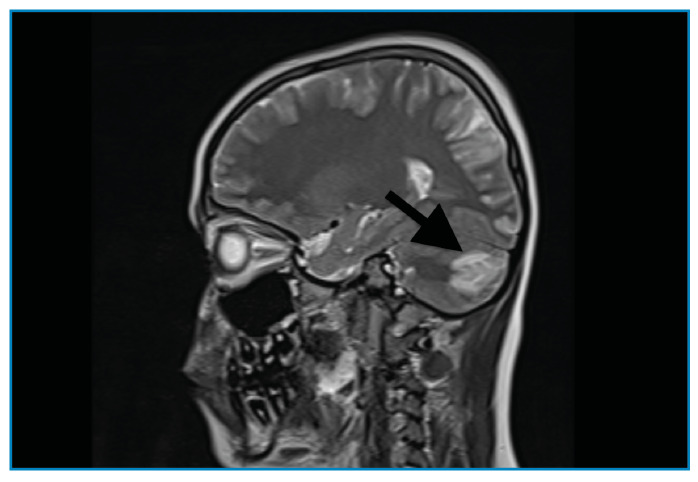
Abnormal signal and contrast enhancement in the right cerebellar hemispheres.

**FIGURE B:**
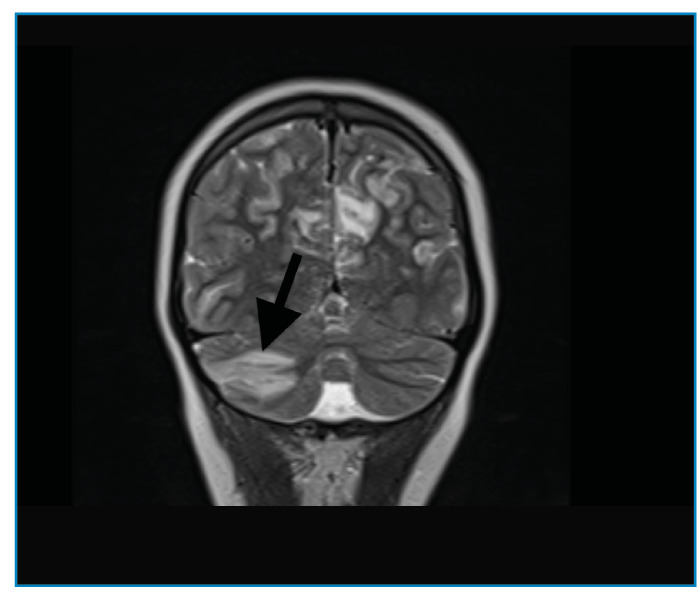
Abnormal signal and contrast enhancement in the right cerebellar hemispheres.

**FIGURE C:**
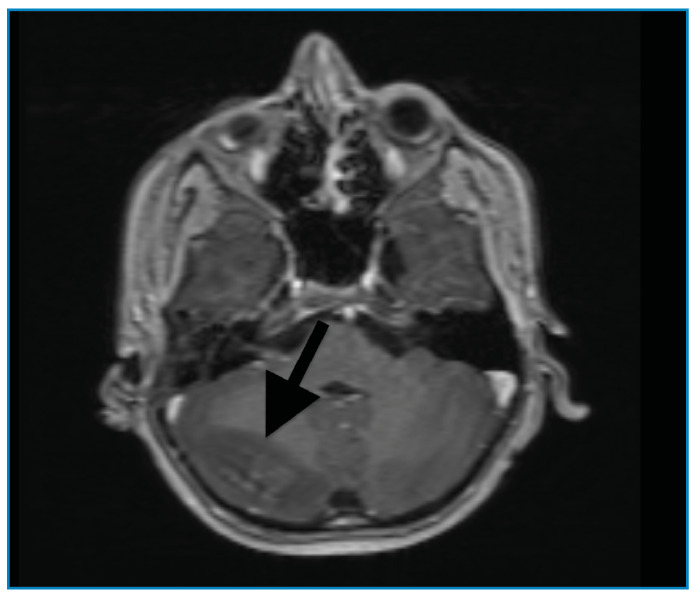
Abnormal signal and contrast enhancement in the right cerebellar hemispheres.
